# Assessing the causal association of pregnancy complications with diabetes and cardiovascular disease

**DOI:** 10.3389/fendo.2024.1293292

**Published:** 2024-06-05

**Authors:** Yuan Xie, Jie Zhang, Shuang Ni, Ji Li

**Affiliations:** ^1^ Department of Gynecology, Longhua Hospital, Shanghai University of Traditional Chinese Medicine, Shanghai, China; ^2^ Central Laboratory for Research, Longhua Hospital, Shanghai University of Traditional Chinese Medicine, Shanghai, China

**Keywords:** gestational diabetes, gestational hypertension, miscarriage, diabetes, cardiovascular disease, Mendelian randomization, causality

## Abstract

**Background:**

To the best of our knowledge, numerous observational studies have linked pregnancy complications to increased risks of diabetes and cardiovascular disease (CVD), causal evidence remains lacking. Our aim was to estimate the association of adverse pregnancy outcomes with diabetes and cardiovascular diseases.

**Methods:**

A two-sample Mendelian randomization (MR) analysis was employed, which is not subject to potential reverse causality. Data for pregnancy complications were obtained from the FinnGen consortium. For primary analysis, outcome data on diabetes, related traits, stroke, and coronary heart disease (CHD) were extracted from the GWAS Catalog, MAGIC, MEGASTROKE, and CARDIoGRAMplusC4D consortium. The MAGIC and UKB consortium datasets were used for replication and meta-analysis. Causal effects were appraised using inverse variance weighted (IVW), weighted median (WM), and MR-Egger. Sensitivity analyses were implemented with Cochran’s Q test, MR-Egger intercept test, MR-PRESSO, leave-one-out (LOO) analysis and the funnel plot.

**Results:**

Genetically predicted gestational diabetes mellitus (GDM) was causally associated with an increased diabetes risk (OR=1.01, 95% CI=1–1.01, *P*<0.0001), yet correlated with lower 2-hour post-challenge glucose levels (OR=0.89, 95% CI=0.82–0.97, *P*=0.006). Genetic liability for pregnancy with abortive outcomes indicated decreased fasting insulin levels (OR=0.97, 95% CI=0.95–0.99, *P*=0.02), but potentially elevated glycated hemoglobin levels (OR=1.02, 95% CI=1.01–1.04, *P*=0.01). Additionally, hypertensive disorders in pregnancy was tentatively linked to increased risks of stroke (OR=1.11, 95% CI=1.04–1.18, *P*=0.002) and CHD (OR=1.3, 95% CI=1.2–1.4, *P*=3.11E-11). Gestational hypertension might have a potential causal association with CHD (OR=1.11, 95% CI=1.01–1.22, *P*=0.04). No causal associations were observed between preterm birth and diabetes, stroke, or CHD.

**Conclusion:**

The findings of this study provide genetic evidence that gestational diabetes, pregnancy with abortive outcomes, and hypertensive disorders in pregnancy may serve as early indicators for metabolic and cardiovascular risks. These insights are pivotal for the development of targeted screening and preventive strategies.

## Introduction

Gestational diabetes mellitus (GDM), hypertensive disorders in pregnancy (HDPs), miscarriage, and preterm birth are prevalent complications during pregnancy. GDM affects approximately 16.7% of live births (1), and HDPs occurs in 2–8% of pregnancies worldwide, posing a significant risk for maternal and perinatal mortality (2). Additionally, the prevalence of miscarriage and preterm labor is 10.8% and 5–18%, respectively (3, 4). Consequently, pregnancy complications exert a considerable burden on global maternal and neonatal health.

While existing research has primarily focused on the short-term consequences of pregnancy complications, including etiology, mechanisms, treatments, and the health outcomes for offspring (5), emerging evidence suggests that pregnancy complications may also have long-term effects on women’s health. Specifically, numerous studies have linked pregnancy complications to a higher risk of developing diabetes (6–8), cardiovascular disease (CVD) (9, 10), and even premature death (11, 12). Diabetes and CVD account for over 70% of global deaths (13). Furthermore, CVD is a leading cause of mortality among women. In 2019, ischemic heart disease and stroke were identified as the major contributors to CVD-related deaths in women (14). Recent findings from the Nurses’ Health Study II have shown that women with a history of spontaneous abortion face a significantly higher risk of premature death, particularly from cardiovascular causes (12). This finding is corroborated by several other studies (15–18).

Despite these associations, there is still a paucity of comprehensive and systematic research appraising the causal relationship between pregnancy complications (abortion, preterm delivery, GDM, HDPs), and the subsequent onset of diabetes and cardiovascular disease. Observational studies are inherent with defects unable to exclude, including potential reverse causality and confounding factors, leading to biased results (19). Furthermore, conducting randomized controlled trials (RCTs) on this subject is both unethical and impractical due to the required resource commitments and long follow-up periods. As such, mendelian randomization (MR) offers a viable alternative for investigating such causal relationships (20). This method utilizes exposure-related genetic variants as instrumental variables (IVs) to explore associations between exposures (e.g., pregnancy complications) and outcomes (e.g., diabetes and CVD) (21). The random assortment of genetic variants at conception prior to disease onset could efficiently minimize the influence of confounding factors, thus providing a more reliable causal inference (22).

Given the above considerations, this study employed genome-wide association study (GWAS) statistics in a two-sample MR framework to rigorously assess the potential causal associations between pregnancy complications and both glycometabolic and cardiovascular disease. Publicly available data from six databases and five sets of diabetes-related traits instruments were used in this study. Additionally, spontaneous delivery cohort served as negative control, replication analysis, and meta-analysis were used to enhance the robustness of MR estimates. Our aim is to provide not only evidence for identification of women at higher risk of diabetes and cardiovascular disease, but also actionable insights for postpartum monitoring and clinical intervention.

## Methods

This Mendelian Randomization (MR) analysis utilized published GWAS summary statistics. The study adheres to the Strengthening the Reporting of Observational Studies in Epidemiology Using Mendelian Randomization (STROBE-MR) guidelines (23).

### Study design

**Figure 1:**
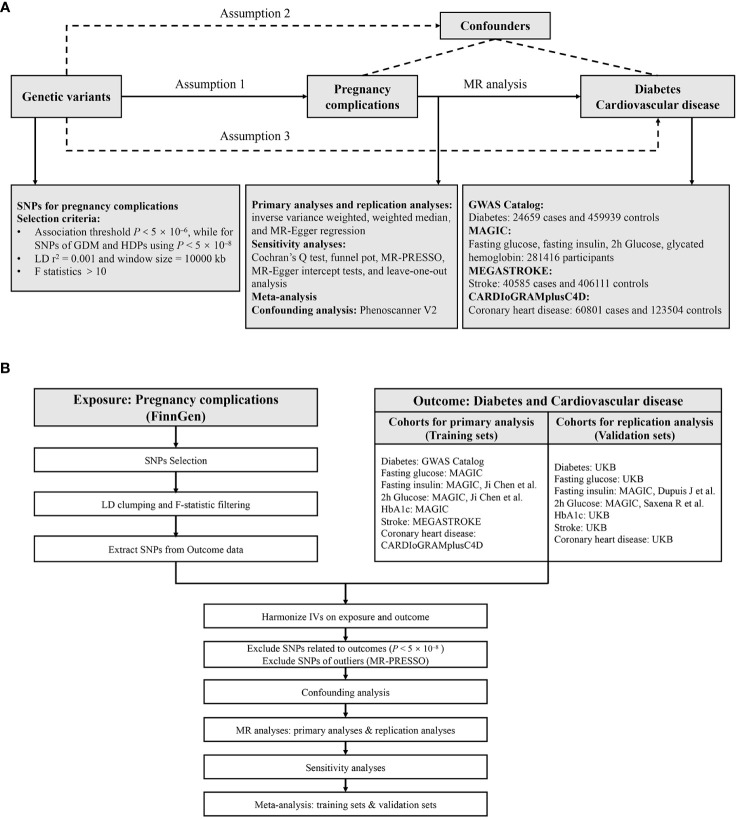
Study design **(A)** and workflow **(B)** of the current MR study. MR, Mendelian randomization; GDM, gestational diabetes mellitus; HDPs, hypertensive disorders in pregnancy; SNPs, single nucleotide polymorphisms; LD, linkage disequilibrium; HbA1c, glycated hemoglobin; UKB, UK Biobank; IVs, instrumental variables; MR-PRESSO, Mendelian randomization pleiotropy residual sum and outlier.

We employed a two-sample MR approach to systematically investigate the causal relationship between pregnancy complications and the risk of diabetes and cardiovascular diseases. A convincing MR design complies with three fundamental assumptions (1): genetic instruments are strongly correlated with exposures (2); these instruments are not associated with confounders (3); the instruments affect the outcome only through the exposure (24). The latter two assumptions are known collectively as independence from horizontal pleiotropy and can be tested through various statistical methods (25). Genetic data for diabetes and cardiovascular diseases were obtained from five independent GWAS consortia for both primary and replication analyses, and then meta-analysis was performed. The study overview is presented in **Figure 1**. All statistical analyses were preformed using the TwoSampleMR package (Version 0.5.7), MRPRESSO (Version 1.0) in R (Version 4.2.2), and Reviewer Manager software (Version 5.3.3).

### GWAS data for pregnancy complications

Six common pregnancy complications, namely gestational diabetes mellitus (GDM), hypertensive disorders in pregnancy (HDPs), gestational hypertension (GH), spontaneous abortion, pregnancy with abortive outcome, and preterm birth were included as exposure in this study. Additionally, spontaneous delivery was included as a negative control cohort to mitigate false positives. Summary-level statistics for these conditions were retrieved from the FinnGen GWAS R9 release (26). The Coordinating Ethics Committee of the Helsinki and Uusimaa Hospital District has approved the FinnGen consortium (Nr HUS/990/2017). Definitions of the above pregnancy outcomes can be found in **Supplementary Table 1**.

### GWAS data for diabetes and cardiovascular diseases

We utilized five sets of instruments indicating different aspects of glucose metabolism and pathophysiology: diabetes, fasting glucose, fasting insulin, 2-hour post-challenge glucose (2hGlu), and glycated hemoglobin (HbA1c). Diabetes is diagnosed as a chronic illness related to dysglycemia and pancreatic impairment. Fasting glucose, 2hGlu, and HbA1c serve as diagnostic indicators for this disease. Moreover, HbA1c is frequently employed as a key biomarker for ongoing glucose control in diabetic patients. Fasting insulin, on the other hand, encapsulates both insulin secretion and insulin resistance, which are integral components in the pathophysiology of diabetes and in insulin clearance (27). Taken together, these four glycemic traits are indispensable for a comprehensive understanding of both diabetes pathophysiology (28–30) and associated cardiometabolic outcomes (31).

The GWAS statistics for diabetes (24659 cases and 459939 controls) in primary analysis were retrieved from GWAS Catalog consortium (32), and SNPs for other markers were obtained from the MAGIC consortium (33). A total of 281416 individuals (~70% European ancestry) without diabetes were included in this study. Data for stroke (40585 cases and 406111 controls) and coronary heart disease (60801 cases and 123504 controls) were obtained from MEGASTROKE and CARDIoGRAMplusC4D consortia, respectively (34). Details of data source are listed in **Table 1**.

**Table 1:** Details of the GWASs included in the Mendelian randomization.

	Trait	Consortium	Case/Control Sample size	Ancestry	PMID
Exposure	Gestational diabetes mellitus	FinnGen	13039/197831	EUR	
	Hypertensive disorders in pregnancy		14727/196143		
	Gestational hypertension		8502/194266		
	Spontaneous abortion		16906/149622		
	Pregnancy with abortive outcome		61248/149622		
	Preterm birth		8507/162777		
	Spontaneous delivery		106627/92306		
Outcome	Diabetes	GWAS Catalog	24659/459939	EUR	33959723
		UKB	5033/189120	EUR	
	Fasting glucose	MAGIC	281416	EUR	34059833
		UKB	167978	EUR	
	Fasting insulin	MAGIC	281416	EUR	34059833
		MAGIC	46186	EUR	20081858
	2h Glucose	MAGIC	281416	EUR	34059833
		MAGIC	15234	EUR	20081857
	Glycated hemoglobin	MAGIC	281416	EUR	34059833
		UKB	185022	EUR	
	Stroke	MEGASTROKE	40585/406111	EUR	
		UKB	1903/192250	EUR	
	Coronary heart disease	CARDIoGRAMplusC4D	60801/123504	EUR (77%) AZN (19%)	26343387
		UKB	2930/191244	EUR	

To corroborate our findings, we conducted replication and meta-analyses using data from the UK Biobank for diabetes, fasting glucose, HbA1c, stroke and CHD, which is publicly available at the website: http://www.nealelab.is/uk-biobank/, and two independent GWAS studies in MAGIC consortium for fasting insulin and 2hGlu (35, 36). More details about the definitions, corresponding ethic committees and approval IDs can be found in **Supplementary Table 2**. GWAS data used in primary analysis is regarded as training sets, while data in replication analysis is validation sets.

### Instruments selection

A multi-step protocol to identify eligible SNPs associated with pregnancy complications were performed. First, given the limited number of SNPs reaching genome-wide significance for gestational hypertension, spontaneous abortion, pregnancy with abortive outcome, preterm birth, and spontaneous delivery, we relaxed the association threshold using *P* < 5 × 10^–6^, while for SNPs of gestational diabetes mellitus and hypertensive disorders in pregnancy using *P* < 5 × 10^–8^. Second, the SNP selection was further refined through linkage disequilibrium (LD) clumping (r^2 =^ 0.001 and window size = 10000 kb) and F-statistic filtering to ensure robust instruments (37). SNPs with F < 10 were recognized as weak instruments and were discarded to ensure all the SNPs conferred sufficient variance for corresponding pregnancy complications (38). Third, we extracted the exposure SNPs from the outcome data and excluded those associated with the outcome (*P* < 5 × 10^–8^). Finally, harmonization was conducted to align the alleles of exposure- and outcome-SNPs, and discard palindromic SNPs with intermediate effect allele frequencies (EAF > 0.42) or SNPs with incompatible alleles (e.g. A/G vs. A/C).

### Primary analyses

The random-effect inverse variance weighted (IVW) method was conducted as the primary analysis to identify significant causal effect with *P* < 0.05. Furthermore, weighted median (WM) and MR-Egger regression were served as supplement to IVW. The weighted median method consistently estimates effects when at least half of the weighted variance remains unaffected by horizontal pleiotropy (39). MR-Egger regression could offer a test for unbalanced pleiotropy and considerable heterogeneity, although it demands a larger sample size for the same underexposure variation (40). Specifically, IVW estimates would be biased if horizontal pleiotropy existed. In this scenario, the MR-Egger estimates should be referenced because this method modifies the IVW analysis to accommodate the potential imbalances or directionalities in horizontal pleiotropic effects across all SNPs (41).

### Sensitivity analyses

Horizontal pleiotropy occurs when genetic variants associated with the exposure of interest (pregnancy complications) directly affect the outcome (diabetes and cardiovascular diseases) through multiple pathways other than the proposed exposure. Therefore, we further conducted Cochran’s Q statistic, funnel plot, Mendelian Randomization Pleiotropy Residual Sum and Outlier (MR-PRESSO), MR-Egger intercept tests and leave-one-out (LOO) analysis to detect the presence of pleiotropy and validate the robustness of the results. Specifically, heterogeneity was detected if the *P* value of the Cochran Q test was less than 0.05. Funnel plots aided in evaluating the probable directional pleiotropy. SNPs with potential pleiotropy were removed after MR-PRESSO, then MR analyses were reperformed to assess robustness. We also appraised horizontal pleiotropy using MR-Egger intercept. To determine whether the causal estimate was driven by any single SNP, we performed LOO analysis, which entails cyclically discarding each exposure-associated SNP to repeat the IVW analysis.

### Replication and meta−analysis

To validate the robustness of results, we replicated mendelian randomization analysis using another independent GWAS data from different consortia mentioned above. This was followed by a meta-analysis to consolidate the results.

### Confounding analysis

Although multiple statistical methods were employed in sensitivity analysis to inspect potential violation of the MR assumptions, we further scrutinized the Phenoscanner V2 website (http://www.phenoscanner.medschl.cam.ac.uk/) to explore whether the exposure-associated SNPs were meanwhile associated with several common risk factors that might bias the MR estimates, including smoking, obesity (42), hypertension (43), and coronary heart disease (44). SNPs associated with these potential confounders at the threshold of *P* < 1×10^–5^ were removed.

## Results

Following the instrument selection steps, F statistics for SNPs after clumping were all over 10, suggesting no weak instruments were employed (**Supplementary Table 3**). The harmonized data of each outcome were presented in **Supplementary Table 4**.

### MR estimates

**Figure 2:**
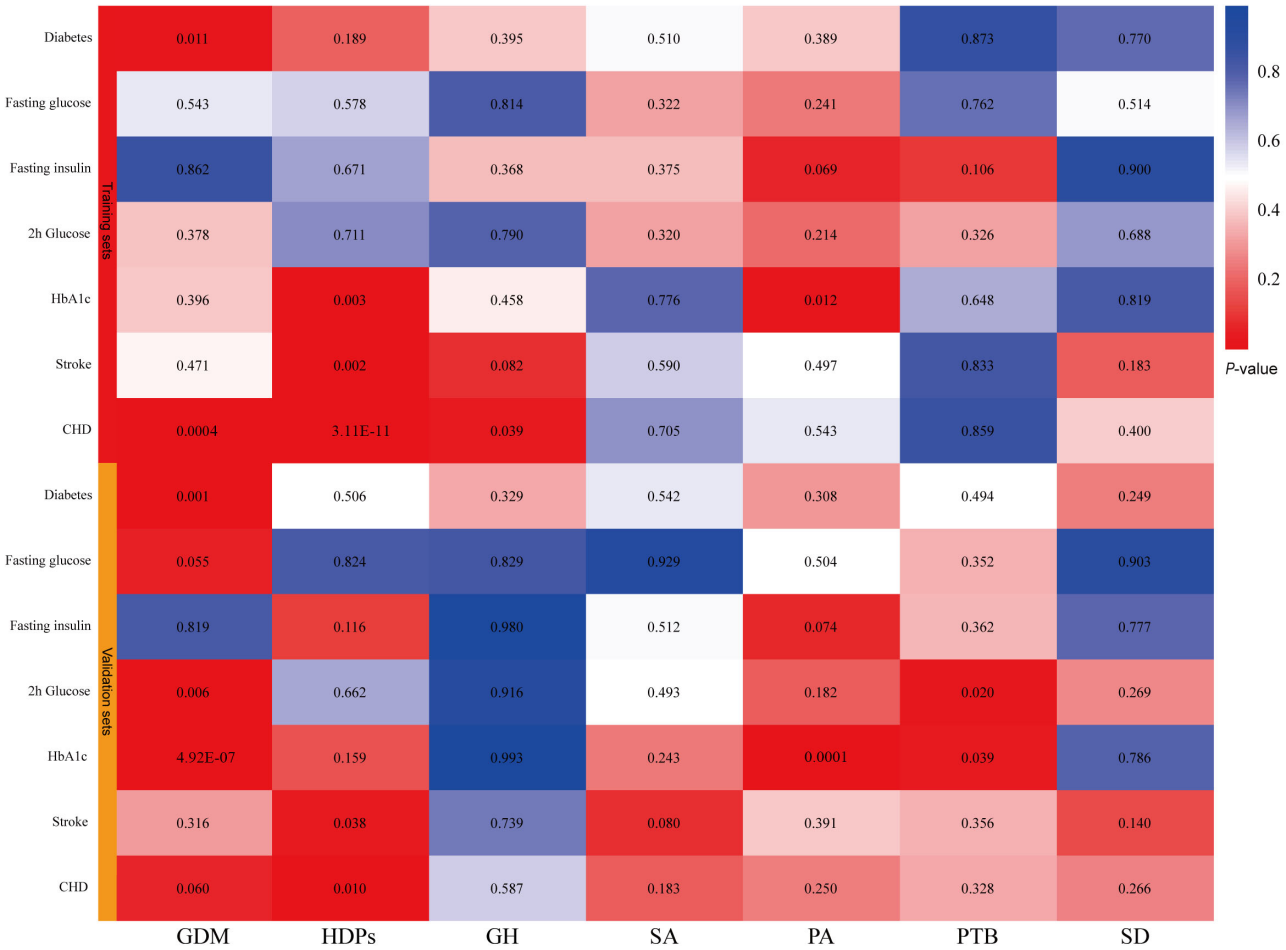
IVW estimates from pregnancy complications on diabetes and cardiovascular disease. The color of each block represents the IVW-derived *P*-values of every MR analysis. *P*-values of < 0.05 were shown in red and *P*-values of > 0.05 were shown in blue. GDM, gestational diabetes mellitus; HDPs, hypertensive disorders in pregnancy; GH, gestational hypertension; SA, spontaneous abortion; PA, pregnancy with abortive outcomes; PTB, preterm birth; SD, spontaneous delivery; HbA1c, glycated hemoglobin; CHD, coronary heart disease.

**Figure 3:**
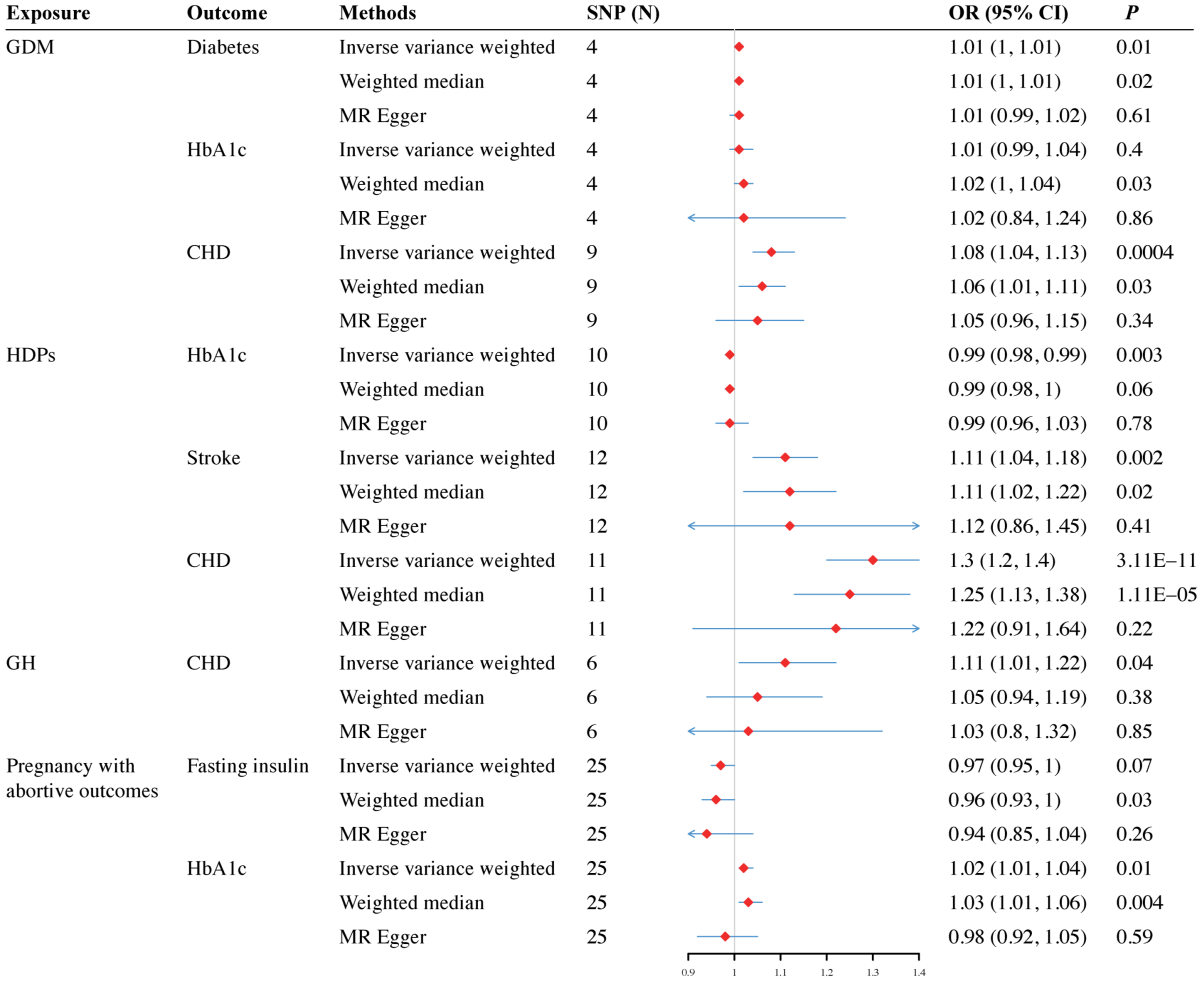
Forest plots for the causal effect of pregnancy complications on the risk of diabetes and cardiovascular disease derived from inverse variance weighted (IVW), weighted median (WM) and MR-Egger analysis. OR, odds ratio; CI, confidence interval; SNPs, single nucleotide polymorphisms; GDM, gestational diabetes mellitus; HDPs, hypertensive disorders in pregnancy; GH, gestational hypertension; HbA1c, glycated hemoglobin; CHD, coronary heart disease.

In the tested gestational diabetes mellitus (GDM) phenotype, IVW analysis indicated that genetically predicted GDM increased the risk of diabetes (OR = 1.01, 95% CI 1–1.01; *P* = 0.01), and coronary heart disease (CHD) (OR = 1.08, 95% CI 1.04–1.13; *P* = 0.0004) (**Figure 2**). The results from other MR methods showed a consistent direction (**Figure 3**). Genetically proxied hypertensive disorders in pregnancy (HDPs) was significantly associated with an increased risk of stroke (OR = 1.11, 95% CI 1.04–1.18; *P* = 0.002), and CHD (OR = 1.3, 95% CI 1.2–1.4; *P =* 3.11E-11) in the IVW analysis (**Figure 2**). Similar causal estimates were obtained from WM model, as in stroke (OR = 1.11, 95% CI 1.02–1.22; *P* = 0.02), and CHD (OR = 1.25, 95% CI 1.13–1.38, *P* = 1.11E-05). Moreover, a positive causality between genetic predisposition toward PA and HbA1c was detected using IVW analysis (OR = 1.02, 95% CI 1.01–1.04; *P* = 0.01) and WM analysis (OR = 1.03, 95% CI 1.01–1.06; *P* = 0.01). A potential causal association of gestational hypertension (GH) with CHD was also observed in the IVW analysis (OR = 1.11, 95% CI 1.02–1.22; *P* = 0.04) (**Figure 3**). However, we did not observe evidence of causal association between preterm birth and diabetes and cardiovascular disease (**Supplementary Table 6**).

### Sensitivity analyses

To assess the robustness of the above results, a series of sensitivity analyses, including Cochran’s Q test, MR-Egger intercept, and MR-PRESSO global test, were conducted (**Supplementary Table 6**). Results of MR-Egger intercept indicated that no pleiotropy existed. However, heterogeneity was observed in the Q test analysis of GDM and diabetes (Q = 10.71, *P* = 0.03), HDPs and CHD (Q = 38.57, *P* = 0.0001). Though heterogeneity was detected, it did not invalidate the MR estimates as random-effect IVW in the current study might balance the pooled heterogeneity. In addition, Egger intercepts did not identify any pleiotropy, suggesting that no pleiotropic bias was introduced to MR estimates in the context of heterogeneity (**Supplementary Figures 1**-**6**). Moreover, LOO analysis revealed that no SNP drove the results, and funnel plots were symmetrical (**Supplementary Figures 7**-**12**), indicating that none of the estimates were violated. Finally, after dropping the outliers detected by MR-PRESSO analysis, causal inference remained in the reperformed MR estimates.

### Replication and meta−analysis

**Figure 4:**
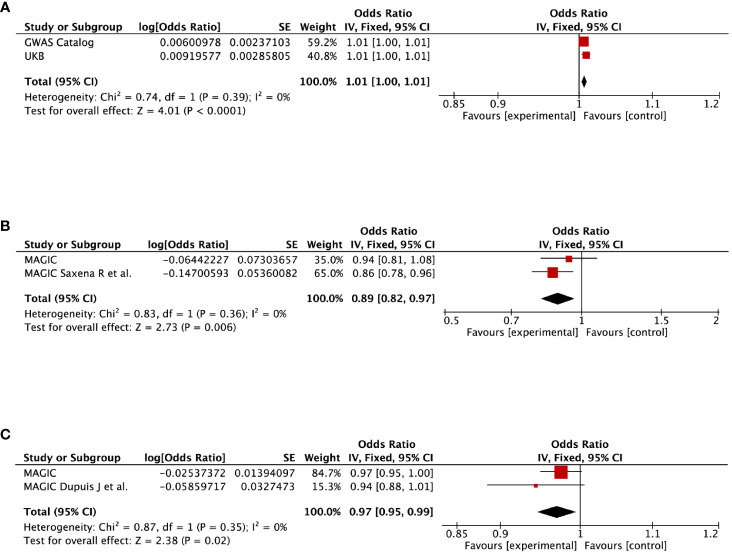
Meta-analysis of the causal impact of **(A)** gestational diabetes mellitus on diabetes, **(B)** gestational diabetes mellitus on 2-hour post-challenge glucose levels, and **(C)** pregnancy with abortive outcomes on fasting insulin levels. CI, confidence interval.

To further verify our results, replication analysis was conducted using independent GWAS data from two different consortia. As expected, similar trends were observed in GDM and diabetes, HDPs and cardiovascular disease (CVD), as well as pregnancy with abortive outcomes and HbA1c (**Supplementary Table 7**). Combined analysis of both datasets identified that genetic liability for GDM predicted a higher risk of diabetes (OR=1.01, 95% CI=1–1.01, *P*<0.0001), but a lower level of 2hGlu (OR=0.89, 95% CI=0.82–0.97, *P*=0.006). Moreover, genetically proxied pregnancy with abortive outcomes predicted a lower level of fasting insulin (OR=0.97, 95% CI=0.95–0.99, *P*=0.02) (**Figure 4**). However, null estimates of the meta-analysis were observed in HDPs and stroke or CHD, as well as pregnancy with abortive outcomes and HbA1c, which attributed to heterogeneity using the validation sets (**Supplementary Figure 19**).

### Confounding analysis

Although bias invalidating the MR estimates had been treated by multiple sensitivity analyses, we further manually investigated the risk factors (smoking, obesity, self-reported hypertension, and coronary artery disease) of the harmonized SNPs. Looking over the Phenoscanner, we found that SNPs associated with spontaneous abortion, GDM and GH were not associated with any of the confounders. For pregnancy with abortive outcomes, two SNPs (rs1898357 and rs2284174) were associated with obesity-related traits. Similar for preterm birth (rs2946160 and rs7591150), and spontaneous delivery (rs7209460 and rs6011779), we identified four SNPs associated with the risk factors mentioned above. The estimates remained after discarding these SNPs.

## Discussion

The present study indicated that genetic predisposition to gestational diabetes mellitus (GDM) was causally associated with an increased risk of diabetes. Furthermore, genetic liability to GDM and pregnancy with abortive outcomes correlated with reduced levels of 2hGlu and fasting insulin, respectively. Additionally, genetic proxied pregnancy with abortive outcomes might lead to a higher level of HbA1c in later life. Genetic liability for hypertensive disorders in pregnancy (HDPs) appeared to be causally related to stroke and coronary heart disease (CHD). Consistent results were obtained in both of the independent datasets, although the meta-analysis results were inconclusive.

Genetic proxied gestational hypertension might have a potential causal impact on risk of CHD. No associations were observed between preterm birth and outcomes of diabetes, stroke, or CHD. To the best of our knowledge, this is the first Mendelian randomization (MR) study using both replication and meta-analysis to systematically evaluate the causal impact of pregnancy complications on diabetes and cardiovascular disease (CVD).

Given the high prevalence and mortality of diabetes and cardiovascular disease, early screening and prevention are of great importance. Previous studies have identified several pregnancy complications as risk factors for diabetes, cardiovascular disease, and even premature mortality. For instance, Wang et al. reported that spontaneous abortion was associated with an increased risk of premature mortality, especially death from cardiovascular disease (12). Moreover, the American Heart Association has classified women with a history of preeclampsia, gestational diabetes, or pregnancy-induced hypertension as being at risk of cardiovascular disease since 2011 (45). However, the existing literature has yet to definitively establish the causal role of pregnancy complications in the onset of diabetes and CVD. Inspired by You et al. (6) and McNestry et al. (46), we designed this multi-exposure MR study to systematically evaluate the causality between pregnancy complications and diabetes, as well as cardiovascular disease (stroke and CHD), aiming to provide robust evidence for risk stratification and targeted screening for women with a history of pregnancy complications.

Our results align with existing literature, suggesting that a genetic predisposition for GDM has detrimental long-term effects, particularly in increasing diabetes risk. In a retrospective study of 321 women with GDM followed up for 1–6 years postpartum, Miao et al. found that insulin resistance and insufficient compensatory insulin secretion remain long-lasting in the postpartum phase, and thus the risk of diabetes is higher (17). During a healthy pregnancy, maternal metabolism undergoes notable shifts, driven primarily by placental hormones. These hormonal changes induce a state of progressive insulin resistance, ensuring that the fetus receives adequate nutrition for optimal growth and development. To compensate for increased insulin resistance, the mother’s β-cells upregulate insulin secretion. However, when this compensatory mechanism is overwhelmed, insulin resistance escalates to prediabetes and eventually leads to a reduction in insulin secretion, culminating in overt diabetes (17). Though specific pathways underlying these metabolic adaptations remain elusive, the parallels between GDM and diabetes are striking. Both conditions are characterized by failed β-cell compensation and insulin insufficiency, suggesting shared pathophysiological mechanisms (47). Recent studies have postulated that pregnancy serves as a metabolic “stress test” for the mother, unmasking latent predispositions to chronic conditions such as diabetes (48). This concept aligns with research suggesting that GDM may be a manifestation of “recessive” diabetes, in which genetic susceptibilities become clinically apparent only under the physiological stress of pregnancy (45). In this way, pregnancy could offer a unique window for early identification and intervention in women at risk for future metabolic and cardiovascular diseases.

Notably, some results deviated from initial expectations. While a higher genetic liability for gestational diabetes mellitus typically suggests impaired glycemic control, our study paradoxically found an association with lower 2hGlu levels. Herein, the MR estimates were consistent across independent datasets and showed no signs of pleiotropy, which also consist with those of previous reports. Riviello et al. demonstrated that postpartum women with overt diabetes, particularly type 1, often require less insulin during lactation and are more prone to hypoglycemia (49). One plausible hypothesis is that lactation enhances pancreatic β-cell adaptability to insulin resistance, thereby stabilizing long-term glycemic control (50). However, this area remains underexplored, underscoring the need for future research. Similarly for pregnancy with abortive outcome, scant literature exists on its relationship with fasting insulin. Existing evidence indicated that threatened miscarriage was inversely correlated with glucose intolerance severity, possibly due to reduced insulin resistance influenced by a lower level of progesterone (51). This was also confirmed in animal experiments (52). Lower fasting insulin levels could also signal compromised β-cell function (53). Further elucidative studies are therefore warranted.

In this study, we performed replication analyses across two independent datasets. Both datasets corroborated that genetic predisposition to pregnancy with abortive outcome was linked with higher HbA1c levels, while genetic liability for hypertensive disorders in pregnancy increased the risk of stroke and coronary heart disease. However, meta-analytic outcomes varied, possibly due to the application of a random-effects model accounting for gender-based heterogeneity. In fact, we could get a positive result if a fixed-effects model was utilized. Though heterogeneity is an unavoidable limitation due to the scarcity of publicly available sex-stratified data, the consistency in our replicated analysis lends credence to our conclusions. Yet, these findings would benefit from further validation through future sex-specific GWAS data. We analyzed gestational hypertension (GH) as a subgroup of hypertension disorders in pregnancy (HDPs). In our study, a potential causal association between genetically predicted GH and coronary heart disease (CHD) was observed only in the training set, with the association being negative in the validation set and meta-analysis. Considering the strong statistical power of the IVW method and the absence of heterogeneity and pleiotropy in sensitivity analyses, we contend that this result should not be ignored. This discrepancy may be due to the relatively small sample size of GH (8502 cases) and CHD (2930 cases) in the validation set. Therefore, our findings warrant validation in future studies with larger sample sizes.

We found no significant associations between preterm birth and diabetes, stroke or coronary heart disease. In contrast, some observational studies suggest such associations (54–56). The discrepancy might arise from residual confounding inherent to observational setting. This is particularly true for cardiovascular disease, which is known to have multifactorial influences (43, 44).

The current study has several strengths. First, a notable strength is the comprehensive selection of exposure and outcome. We incorporated double cohorts (spontaneous abortion and pregnancy with abortive outcome) related to abortion and a negative cohort (spontaneous delivery) for MR analysis. By utilizing five sets of instruments, we provided an in-depth view of glycemia metabolic function, making our study a systematic exploration of pregnancy complications’ contribution to diabetes and cardiovascular disease. Second, the MR design mitigates concerns about reverse causation and residual confounding. We employed multiple MR and sensitivity analyses to confirm adherence to MR assumptions, enhancing the validity of our estimates. Third, application of replication and meta-analyses further strengthen the study. The consistent findings across datasets validate the robustness of our results.

Several limitations should be noted in our study. First, given the limited SNPs for spontaneous abortion, pregnancy with abortive outcome, preterm birth, and spontaneous delivery reaching genome-wide significance, we relaxed the *P* threshold, which is a widely accepted approach (57). However, the F statistic for these SNPs exceeded 10, suggesting no weak instruments were included. Second, the study predominantly features European participants. While this minimizes population heterogeneity, it necessitates further validation in diverse ancestries to ensure broad applicability. Third, although we examined multiple exposures, we did not adjust MR estimations for multiple testing. Instead, we opted for replication analysis with independent datasets, enhancing the credibility. We contend that an overly conservative multiple testing threshold might overshadow individually significant associations. Furthermore, due to the lack of GWAS data that exclusively includes patients with hypertensive disorders in pregnancy (HDPs), the interpretation of the results considering HDPs in our study needs to be cautious. Finally, although we used sex-specific data for exposure, outcome data were largely pooled from GWAS statistics for both genders due to the scarcity of gender-stratified data in public repositories, which is inevitable in similar studies (58).

## Conclusions

In conclusion, gestational diabetes was causally associated with an increased risk of diabetes but a lower level of 2hGlu. Pregnancy with abortive outcomes predicted a lower level of fasting insulin but probably a higher level of HbA1c in later life. In addition, hypertensive disorders in pregnancy were probably causally associated with a higher risk of stroke and coronary heart disease (CHD). However, no association were noted between preterm birth and the outcome of diabetes, stroke or CHD. Our findings underscore the clinical importance of pregnancy as a critical period for identifying women at higher risk for diabetes and cardiovascular disease, thereby informing targeted risk stratification and early interventions.

## Data availability statement

The datasets presented in this study can be found in online repositories. The names of the repository/repositories and accession number(s) can be found in the article/**Supplementary Material**.

## Ethics statement

The studies involving humans were approved by corresponding ethics committee. The studies were conducted in accordance with the local legislation and institutional requirements. Written informed consent for participation was not required from the participants or the participants’ legal guardians/next of kin in accordance with the national legislation and institutional requirements.

## Author contributions

YX: Conceptualization, Data curation, Investigation, Methodology, Writing – original draft, Writing – review & editing. JZ: Conceptualization, Formal Analysis, Visualization, Writing – review & editing, Funding acquisition. SN: Conceptualization, Visualization, Writing – review & editing. JL: Conceptualization, Funding acquisition, Supervision, Writing – review & editing.
