# Supplementary material for: Assessing the causal association of pregnancy complications with diabetes and cardiovascular disease
Source: Front Endocrinol (Lausanne). 2024 Jun 5;15:1293292. doi: 10.3389/fendo.2024.1293292 (PMC11188328; doi:10.3389/fendo.2024.1293292)
Supplement: Supplementary file 2 [file DataSheet_2.docx]

Supplementary Material

**Figure S7.** Leave-one-out sensitivity analysis. (A-G) Exposures (GDM, HDPs, GH, SA, PA, PTB, SD) and diabetes; (H-I) Exposures (GDM, HDPs) and fasting glucose. GDM, gestational diabetes mellitus; HDPs, hypertensive disorders in pregnancy; GH, gestational hypertension; SA, spontaneous abortion; PA, pregnancy with abortive outcome; PTB, preterm birth; SD, spontaneous delivery.

**Figure S8.** Leave-one-out sensitivity analysis. (A-E) Exposures (GH, SA, PA, PTB, SD) and fasting glucose; (F-I) Exposures (GDM, HDPs, GH, SA) and fasting insulin.

**Figure S9.** Leave-one-out sensitivity analysis. (A-C) Exposures (PA, PTB, SD) and fasting insulin; (D-I) Exposures (GDM, HDPs, GH, SA, PA, PTB) and 2-hour post-challenge glucose.

**Figure S10.** Leave-one-out sensitivity analysis. (A) SD and 2-hour post-challenge glucose; (B-H) Exposures (GDM, HDPs, GH, SA, PA, PTB, SD) and glycated hemoglobin; (I) GDM and stroke.

**Figure S11.** Leave-one-out sensitivity analysis. (A-F) Exposures (HDPs, GH, SA, PA, PTB, SD) and stroke; (G-I) Exposures (GDM, HDPs, GH) and coronary heart disease.

**Figure S12.** Leave-one-out sensitivity analysis. (A-D) Exposures (SA, PA, PTB, SD) and coronary heart disease.
