# Supplementary figures and images for: Assessing the causal association of pregnancy complications with diabetes and cardiovascular disease
Source: Front Endocrinol (Lausanne). 2024 Jun 5;15:1293292. doi: 10.3389/fendo.2024.1293292 (PMC11188328; doi:10.3389/fendo.2024.1293292)

Supplementary Material


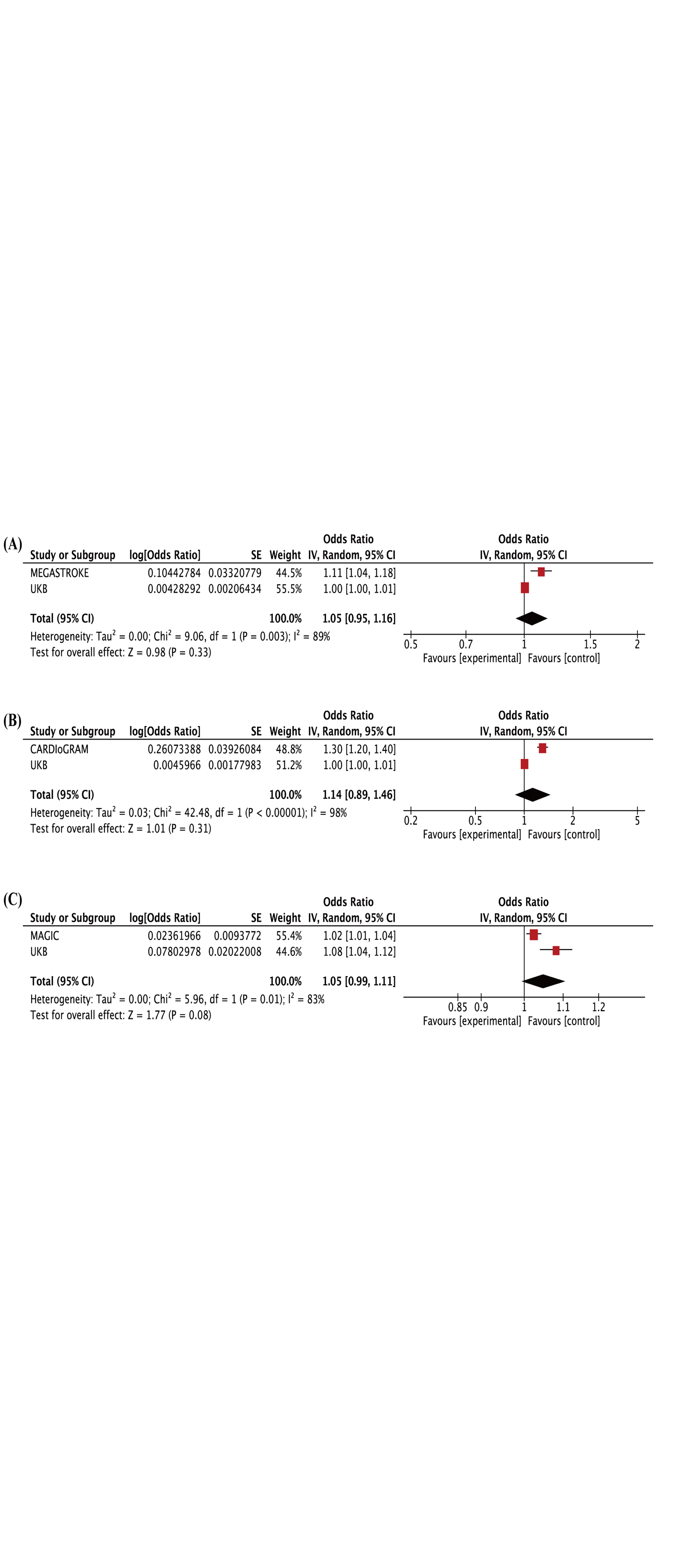


Figure S19. Meta-analysis of (A)

Supplement: Supplementary file 4 [file DataSheet_4.docx]
